# Structural analysis of clinically relevant pathogenic G6PD variants reveals the importance of tetramerization for G6PD activity

**DOI:** 10.19185/matters.201705000008

**Published:** 2017-09-14

**Authors:** Anna D Cunningham, Daria Mochly-Rosen

**Affiliations:** Department of Chemical and Systems Biology, Stanford University School of Medicine; Department of Chemical and Systems Biology, Stanford University School of Medicine

**Keywords:** G6PD, Protein Multimerization, Structure–Activity Relationship

## Abstract

Over 220 different amino acid variants have been identified in human glucose-6-phosphate dehydrogenase (G6PD), covering over 30% of the protein sequence. Many of these variants are pathogenic, causing varying degrees of G6PD deficiency with symptoms ranging from severe chronic anemia (class I) to milder triggered hemolytic episodes (classes II and III). The phenotypic effects of most G6PD variants have been reported, providing an opportunity to correlate phenotypic and structural information. In particular, we sought to investigate the tetramer interface of G6PD in relation to pathogenic variation, as there are conflicting reports indicating the importance of tetramerization for G6PD activity. Using a three-dimensional spatial scan statistic, hotspots of structural enrichment were identified for each class of pathogenic G6PD variants. Class I variants, the most phenotypically severe, were enriched at the dimer interface, consistent with previous evidence that dimerization is essential for G6PD activity. Class II variants were enriched near the tetramer interface, suggesting that tetramerization is also important for G6PD activity. This analysis explains why these two classes, both yielding 10% or less G6PD activity as compared to normal, lead to different clinical outcomes.

## Objective

The objective of this study is to infer the functional roles of G6PD structural regions, specifically dimerization and tetramerization, by examining the enrichment or depletion of pathogenic and benign G6PD variants in and near these regions.

## Introduction

Glucose-6-phosphate dehydrogenase (G6PD) is observed in both dimeric and tetrameric forms, but the importance of the dimer–tetramer equilibrium for G6PD activity is not fully understood. Monomeric G6PD is inactive [1] [2]; however, no kinetic distinction between G6PD dimers and tetramers has been reported [3] [4]. Previous studies suggest that tetramerization protects G6PD from inactivation by NADPH [2], whereas a more recent study concluded that G6PD dimerization is sufficient for activity and that thus the tetramer state is not required [5]. Here we investigated the importance of G6PD tetramerization by determining whether amino acid variants that are likely to affect tetramerization also cause clinical pathology.

Over 160 identified human variants in G6PD lead to G6PD deficiency [6], which is categorized into tiers based on clinical severity: class I (<10% activity and chronic anemia), II (<10% activity and triggered hemolytic episodes), III (10–60% activity and triggered hemolytic episodes), or IV (>60% activity and asymptomatic) [7] [8]. Additionally 64 G6PD variants of unknown clinical consequence have been reported in a reference population database [9] [10]. This large number of G6PD variants, coupled with detailed clinical stratification, allows us to robustly identify three-dimensional regions statistically enriched in different classes of variants [11], and thus infer structure–function relationships in G6PD.

## Results & Discussion

**Figure 1:**
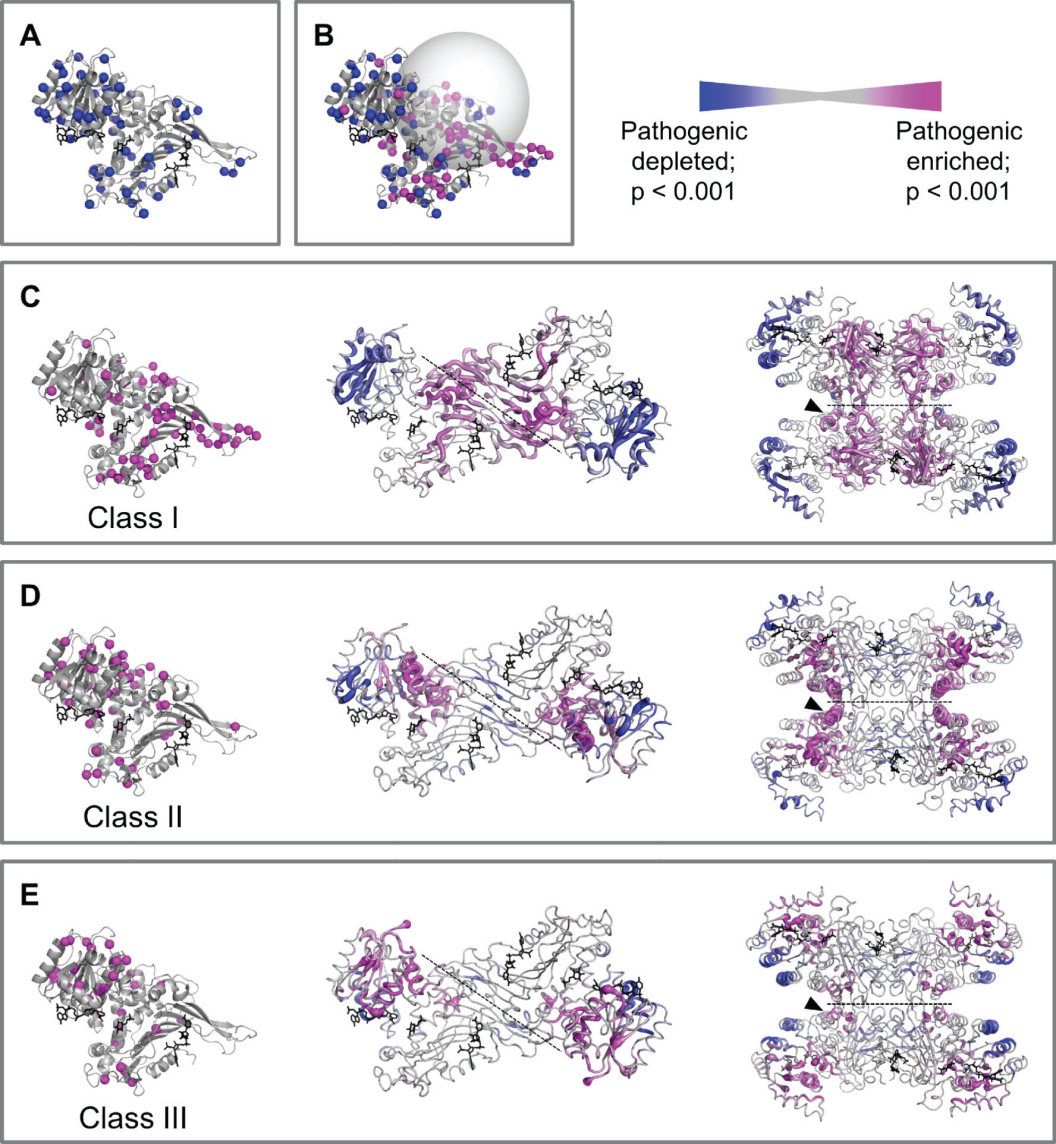
**(A)** The locations of the variants from the reference population database (ExAC), designated as class IV (benign) for the spatial scan statistic calculation. **(B)** An example of a sphere used for calculating the spatial scan statistic at amino acid position 213, which was the most enriched sphere for class I variants. **(C–E)** The locations of G6PD variants are shown in spheres on the monomeric crystal structure (left), and the spatial scan statistic is represented on the dimeric (middle) and tetrameric (right) structures, for **(C)** class I variants, **(D)** class II variants, or **(E)** class III variants, respectively. Color represents the value of the spatial scan statistic (magenta: enrichment of pathogenic variants compared to benign variants; blue: depletion of pathogenic variants compared to benign variants). Thickness of the cartoon backbone represents *p*-value. Structural NADP^+^ is shown in black sticks. Dotted lines indicate the dimer (middle) and tetramer (right) interfaces. A black arrow marks the alpha -helix at the tetramer interface. PDB: 1QKI.

Recently, Homburger et al. used a spatial scan statistic to identify three-dimensional protein regions that are enriched in pathogenic variants and depleted in benign variants [11]. Following their example, we classified human G6PD variants found in the reference population [9] [10] as benign (figure 1A). In addition, many of these variants were predicted to be benign by two different variant prediction algorithms, PolyPhen2 and SIFT [9].

Briefly, the spatial scan statistic is calculated by defining a sphere (we used a radius of 15 Å) centered at each residue in the protein’s crystal structure and then comparing the number of pathogenic and benign variants inside the sphere to the number of pathogenic and benign variants outside the sphere [11] (figure 1B). To capture patterns of variation across oligomeric interfaces, we used the tetrameric structure of G6PD -(PDB: 1QKI) [4]. We calculated the spatial scan statistic three times, using either class I (<10% G6PD activity, severe phenotype), class II (<10% activity, mild phenotype), or class III (10–60% activity, mild phenotype) as pathogenic variants. (Spatial scan statistic values and *p*-values are included in the supplementary data.) The statistic was mapped onto the 3D structure of G6PD using a colored scale for ease of visualization (figure 1C–E).

We observed the strongest enrichment of class I variants around the dimer interface and structural NADP^+^ binding site (figure 1C), which is an allosteric site important for the stability of G6PD. Conversely, class II and class III variants were depleted in the dimer interface (figure 1D–E, middle), thus confirming that dimerization of G6PD is essential for activity because variants disrupting dimerization lead to the most severe loss of activity and clinical phenotype of chronic anemia (class I).

Class II variants were strongly enriched in an alpha -helix that supports and partially forms the tetramer interface (figure 1D, right), whereas class III variants were not enriched in this region (figure 1E, right). This means that variants at or near the tetramer interface, which likely disrupt tetramerization, result in a more severe G6PD deficiency phenotype (class II as compared with class III). We conclude that disruption of tetramerization leads to very low (<10%) G6PD activity, and therefore tetramerization is important for G6PD activity. Based on the previous finding that tetramerization protects G6PD against inhibition by NADPH, it is possible that variants that disrupt tetramerization may allow NADPH-induced inhibition of G6PD activity, leading to <10% G6PD activity and a class II G6PD deficiency phenotype. Additionally, although G6PD dimerization is sufficient for catalytic activity *in vitro* [5], it is possible that tetramerization improves catalytic efficiency and/or stability of the enzyme under physiological conditions. (Note that protein stability is particularly important in anucleate erythrocytes that have a half-life of 100–120 days and minimal or no *de novo* synthesis.)

Class III variants were enriched in the catalytic domain, peripheral to and not directly overlapping with the catalytic pocket. This suggests that class III variants reduce catalytic activity by directly affecting the conformation of the catalytic pocket and likely have few other structural or allosteric consequences, leading to a mild clinical phenotype.

This analysis also provides a structural explanation for why class I and II variants, which are defined by the same activity range (<10% compared to normal), lead to different clinical outcomes: class I causing chronic anemia and class II causing episodic, trigger-induced anemia. As previously shown, class I variants generally have low protein stability whereas class II variants generally have low catalytic activity [9] [12]. This analysis reveals the structural elements that likely contribute to these different biochemical properties: the structural NADP^+^ binding site and dimer interface, which are enriched in class I variants, likely contribute to protein stability, whereas the tetramer interface, which is enriched in class II variants, likely contributes to catalytic activity.

## Conclusions

By examining the structural distributions of different classes of G6PD variants, we have shown that severely pathogenic (class I) variants are enriched at the dimer interface, confirming the importance of dimerization in G6PD activity. Class II variants (leading to <10% enzyme activity) are enriched at the tetramer interface, suggesting that tetramerization plays an important role in G6PD activity and shedding new light on conflicting reports of G6PD tetramerization in relation to enzyme activity. Class III variants (leading to 10–60% activity) are enriched in the catalytic domain but exclude the catalytic site *per se*, suggesting that class III variants reduce activity by directly affecting the catalytic pocket.

Taken together, by correlating phenotypic effects with structural variation, this study reveals how various structural regions of G6PD contribute to the function of the enzyme.

## Limitations

A central assumption when mapping variants to the three-dimensional structure is that these variants do not grossly affect the folding or conformation of the monomeric enzyme. However, a variant that disrupts folding or protein conformation would likely have a severe effect on the function of G6PD and thus would likely be embryonically lethal or cause a class I phenotype. Therefore, although variants may grossly disrupt the enzyme conformation and complicate the conclusions drawn in this study, this possibility is likely to only affect the analysis of the class I variants, and not class II or III. For example, the published crystal structure of Canton G6PD (R459L; PDB: 1QKI; [4]), a class II variant, does not differ substantially from the wild-type crystal structures (PDB: 2BH9, 2BHL; [13]).

## Supplementary Material

Class I-III, Variants
